# Covalent Organic Framework (C_6_N_6_) as a Drug Delivery Platform for Fluorouracil to Treat Cancerous Cells: A DFT Study

**DOI:** 10.3390/ma15217425

**Published:** 2022-10-22

**Authors:** Mohammed A. Alkhalifah, Muhammad Yar, Imene Bayach, Nadeem S. Sheikh, Khurshid Ayub

**Affiliations:** 1 Department of Chemistry, College of Science, King Faisal University, Al-Ahsa 31982, Saudi Arabia; 2 Department of Chemistry, COMSATS University Islamabad, Abbottabad Campus, Abbottabad 22060, KPK, Pakistan; 3 Chemical Sciences, Faculty of Science, Universiti Brunei Darussalam, Jalan Tungku Link, Gadong BE1410, Brunei

**Keywords:** drug delivery, cancer, covalent triazine framework C_6_N_6_, density functional theory

## Abstract

Continuous studies are being carried out to explore new methods and carrier surfaces for target drug delivery. Herein, we report the covalent triazine framework C_6_N_6_ as a drug delivery carrier for fluorouracil (FU) and nitrosourea (NU) anti-cancer drugs. FU and NU are physiosorbed on C_6_N_6_ with adsorption energies of −28.14 kcal/mol and −27.54 kcal/mol, respectively. The outcomes of the non-covalent index (NCI) and quantum theory of atoms in molecules (QTAIM) analyses reveal that the FU@C_6_N_6_ and NU@C_6_N_6_ complexes were stabilized through van der Waals interactions. Natural bond order (NBO) and electron density difference (EDD) analyses show an appreciable charge transfer from the drug and carrier. The FU@C_6_N_6_ complex had a higher charge transfer (−0.16 e^−^) compared to the NU@C_6_N_6_ complex (−0.02 e^−^). Frontier molecular orbital (FMO) analysis reveals that the adsorption of FU on C_6_N_6_ caused a more pronounced decrease in the HOMO-LUMO gap (E_H-L_) compared to that of NU. The results of the FMO analysis are consistent with the NBO and EDD analyses. The drug release mechanism was studied through dipole moments and pH effects. The highest decrease in adsorption energy was observed for the FU@C_6_N_6_ complex in an acidic medium, which indicates that FU can easily be off-loaded from the carrier (C_6_N_6_) to a target site because the cancerous cells have a low pH compared to a normal cell. Thus, it may be concluded that C_6_N_6_ possesses the therapeutic potential to act as a nanocarrier for FU to treat cancer. Furthermore, the current study will also provide motivation to the scientific community to explore new surfaces for drug delivery applications.

## 1. Introduction

Cancer is a leading cause of death worldwide and poses a serious threat to public health. To deal with this daunting disease, several therapies, such as chemotherapy, hormone therapy, and radiotherapy [1] are in use. However, these therapies have several associated side effects. Some of the common side effects are breast and throat swelling, anemia, blurred vision, pain, nausea, hair loss, fatigue, skin and mood changes, stomach bloating, and vaginal bleeding [2]. Furthermore, the application of diverse antineoplastic drugs has been explored to evaluate their efficacy to destroy cancerous cells [3,4]. Undoubtedly, some of the extensively used anti-cancerous drugs in the pharmaceutical sector include nitrosourea (NU), 5-fluorouracil (FU), and their derivatives [5,6,7,8,9]. Despite their remarkable therapeutic properties, normal cells are prone to harmful effects if these drugs are used at high concentrations. This has been reported for the conventional chemotherapeutic methods, which suffer from a lack of specificity and lead to the destruction of normal cells along with melanotic cells [10,11]. Considering this, it is vital to efficiently control the plasma concentration of antineoplastic drugs via an effective and targeted drug delivery system (DDS). The dosage of anti-cancerous drugs can be adjusted according to the given circumstances, including the severity of the disease and the patient’s tolerance level in targeted DDS [12,13,14].

Owing to the significance of DDSs in medicinal chemistry, the development of innovative and highly effective DDSs with improved therapeutic profiles is inevitable. Arguably, the application of DDSs has gained enormous attention as one of the most promising and efficient methods reported for the treatment of cancer and which abates the severely harmful side effects of the conventional approaches to cancer treatment [15]. One of the key features of DDSs involves the administration of a controlled dosage of drugs to target the tumor cells without noticeable degradation of the drug molecules [16,17,18,19]. Among the various approaches that are used for DDS, chronopharmacology is one of them. With chronopharmacology, the drug is delivered to a patient through a staggered profile system [20]. Subsequently, the protonation of the drug molecule influenced by the acidic environment of the cancerous tissues weakens the binding interaction of the drug with the carrier and results in an effective off-loading of the drug molecule from the carrier to the cancerous tissues [21]. As expected, this therapeutic protocol is thwarted by some serious challenges, including poor absorption and compromised biocompatibility of the drug molecules on the carrier surface [22].

Gratifyingly, recent progress in the field of nanotechnology has revolutionized almost every aspect of research and has substantially contributed to the diagnosis and treatment of several deadly diseases. In addition, controlled drug delivery has been carried out both experimentally and numerically. In a reported study, the controlled drug release of gliclazide from a polymeric matrix system was studied [23]. Numerous reports have highlighted the selective attack of nanoparticles on cancerous cells without producing toxic effects on normal cells [24,25,26]. Due to the extensive research executed in this area, the nanoparticle-based drug delivery system (NDDS) has emerged as a highly efficient, well-controlled, and target-oriented protocol [27]. Moreover, the NDDS reveals an enormous potential in cancer treatment by providing a reasonable solution to the poor absorption problem [9] and has demonstrated several advantages, including a prolonged half-life, better bio-distribution, controlled and preserved drug release, a better circulation time of the drug, and versatility in the modes of administration [28,29,30,31]. Extracellular vesicles, which naturally carry endogenous bioactive nucleic acids, are also capable of transferring small interfering RNAs to the target cancer cells [32]. In addition, a large number of near-infrared light-sensitive drug delivery systems have been successfully devised, which have showcased promising results for the treatment of cancer [16].

The ubiquitous presence of computational investigation to better understand and elaborate the research findings is beyond any doubt, and the field is attracting serious attention from the scientific community day by day. In-depth theoretical studies have played a pivotal role in the development of DDSs as well, and their contribution is on the rise. For example, Samanta et al. explored the application of a fullerene (C_60_) surface for temozolomide and carmustine and reported that the release of these chemotherapeutic drugs to biological systems is considerably facilitated by the enhanced polarity of C_60_ when attached to these anti-cancer drugs [33]. Mechanistically, several factors contribute to a facile release of drugs that are primarily dependent on the nature of the drug carrier, type of affinity (either chemical or physical), and the morphological features of the drugs under investigation. Some of the commonly reported mechanisms associated with the release of drug molecules from nanocarriers highlight the role of diffusion, solvent, chemical reaction, and stimuli-controlled processes [34,35,36]. Over the past decade, different types of NDDS have been reported that demonstrate the use of silver, gold [37], iron oxide [38], dendrimers [39], polymeric micelles [40,41], liposomes [42,43], quantum dots [44], and carbon nanotubes [45,46] as nanocarriers for the drug molecules. At the present time, ultrathin two-dimensional materials, such as graphene and its derivatives (graphene oxide, reduced graphene), transition metal dichalcogenides, hexagonal boron nitride, Mxenes (monolayers of Si, Ge, and Sn), and phosphorenes, are extensively used NDDSs due to their remarkable mechanical, electrical, and optical properties, in addition to their enhanced surface area [47,48,49]. In general, 2D materials exhibit great potential for drug delivery due to a high surface area for drug loading [50,51,52,53]. There have been several reports in the literature that shed light on the successful application of 2D materials in DDSs. Yang et al., for instance, theoretically investigated the use of phosphorene and hexagonal boron nitride nanosheets as DDSs for fluorouracil and mercaptopurine, respectively [32]. More recently, Mohammed et al. demonstrated the potential of pristine graphene and metal-doped graphene nanosheets as DDSs for various potent anti-cancer drugs, including 6-mercaptopurine cyclophosphamide and fluorouracil [33]. Another DFT investigation highlighted the use of fluorinated graphene oxide as an efficient carrier for doxorubicin and camptothecin, similar to a study that reported on the use of silver- and gold-coated iron nanoparticles (for the delivery of mercaptopurine and cisplatin) and silicone oxide nanoparticles (for the delivery of gemcitabine) [28,54].

As detailed above, the fundamental and challenging issues faced during the delivery of anti-cancer drugs with macrosystems are poor absorption and poor biocompatibility in specific regions due to the lack of the optimal adsorption of the drug molecule onto the surface of the drug carrier. Nanostructures that have been studied experimentally for drug delivery systems are primarily metal alloys and quantum dots, which have low biocompatibility due to their hydrophobic nature [55]. Carbon nitrides were recently reported to have a mix of hydrophobic and hydrophilic natures, with a high surface-to-volume ratio, which is helpful for finding their applications in drug delivery systems. Among these, carbon nitride, C_6_N_6_, is the one with the highest nitrogen contents and is expected to have quite good hydrophilicity. Therefore, we became interested in studying the application of C_6_N_6_ for drug delivery. Moreover, high nitrogen contents can provide good binding of the drug molecules to the C_6_N_6_ surface. Owing to our continuous interest in the development of an efficient drug delivery system for anti-cancer drug molecules [55,56], herein, we report our investigation of a two-dimensional covalent triazine framework (C_6_N_6_) as a drug delivery platform for NU and FU. The reports in the literature have revealed that C_6_N_6_ has not yet been investigated as a DDS for the FU and NU anti-cancerous drugs. Our proposed system can effectively interact with the targeted drugs because of its unique characteristics related to an electron-rich cavity and high surface area [57]. The current study theoretically examined the nature of the interactions between anti-cancerous drugs FU and NU with C_6_N_6_, which are commonly used for cancer treatments. DFT simulations have been applied to study the mechanism of drug delivery and to provide a detailed investigation of the electronic properties of this class of materials, as well as a better understanding of the nature of the interactions between the studied drug molecules and the triazine framework.

## 2. Computational Methodology

All the simulations in the current study were carried out by employing Gaussian 09 software. The geometries of the C_6_N_6_ and Drug@C_6_N_6_ complexes were computed at a ωb97XD/6-31++G (d, p) level of theory. ωb97XD is range-separated functional that is considered best for non-covalent interactions [56]. In ωb97XD, the ω is a function of range-separation, which efficiently reduces self-interaction error [57]. Moreover, ωb97XD includes Grimme’s D2 dispersion model to effectively capture van der Waals interactions [57]. The electronic properties, such as frontier molecular orbitals (FMO), the natural bond order (NBO), and the electron density difference (EDD) for all the complexes were performed at the same level of theory [58,59,60,61]. Drug molecules are adsorbed on C_6_N_6_ by different orientations to find the most stable geometry of each complex. Frequency analysis was carried out to confirm the true minimum nature of each optimized complex on the potential energy surface. The adsorption energies of Drug@C_6_N_6_ were calculated as:

∆E = [E_(complex)_ − (E_C6N6_ + E_Drug_)]

where E_complex_ is the energy of the Drug@C_6_N_6_ complex_,_ E_C6N6_ is the energy of C_6_N_6_, and E_Drug_ is the drug molecule. The nature of the interactions between the drugs and C_6_N_6_ was explored by non-covalent interaction (NCI) analysis. NCI analysis mainly comprises a reduced density gradient (RDG), the product of the electron density (ρ), and the sign of λ_2_. The RDG is a product of the density and its derivative, which is represented as [62].

$$s=\frac{1}{2{\left(3\pi^{2}\right)}^{\frac{1}{3}}}\;\frac{\nabla \rho }{\rho^{4/3}}$$

In non-covalent interactions, the electron density (*ρ*) is small. Thus, a small change in density gives a noticeable change in the RDG scale. The strength and nature of non-covalent interactions are explored through product (λ_2_) *ρ*. So, the nature of the NCI depends on the sign and value of λ_2_. If the product of (λ_2_) *ρ* is large and negative, electrostatic interactions appear in the form of blue spikes in low RDG regions. If (λ_2_) *ρ* is negative and small (below −0.02 a.u.), NCIs are projected in the form of green spikes, which indicate the presence of van der Waals interactions. The large and positive values of (λ_2_) *ρ* show repulsive interactions and are projected in the form of red spikes [63,64,65].

Quantum theory of atoms in molecules (QTAIM) analysis provides detailed information about inter- and intra-molecular interactions. Through QTAIM analysis, various types of topological terms, such as bond critical points (BCPs), ring critical points (RCPs), cage critical points (CCPs), and nuclear critical points (NCPs) can be explored. Bond critical points are usually applied to describe the nature of NCIs. Various parameters, such as the electron density (*ρ*), the Laplacian of the electron density ($\nabla^{2}$*ρ*), the potential energy density (V), the local kinetic energy density (G), and the electronic energy density (H) are used to explain the BCPs [66]. The interactions are covalent in nature when *ρ* is greater than 0.1 a.u. and ($\nabla^{2}$*ρ*) is large and negative. On the other hand, *ρ* < 0.1 a.u. and a positive ($\nabla^{2}$*ρ*) value reveals close shell interactions [67,68]. Multiwfn 3.7 software was employed for the NCI, QTAIM, and EDD analyses [69]. The dipole moment and pH effects were also studied for the drug delivery of FU and NU at target sites by the C_6_N_6_ carrier.

## 3. Results and Discussion

### 3.1. Geometry Optimization and Interaction Energy

**Figure 1:**
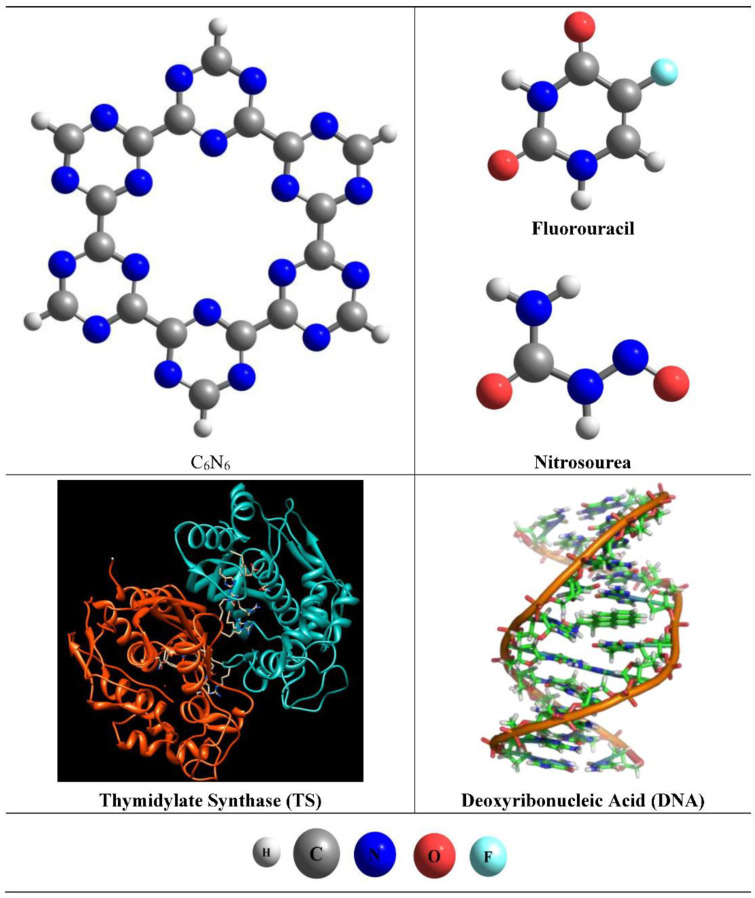
Optimized geometries of cluster model of C_6_N_6_, fluorouracil, and uracil at ωb97XD/6-31++G (d,p) level of theory. General representation of anti-cancerous drug receptors thymidylate synthase (TS) and deoxyribonucleic acid.

Initially, we optimized the cluster model of C_6_N_6_ through DFT simulations (see Figure 1). Bond distances of 1.53 Å and 1.33 Å were observed for the C-C and C-N bonds of the C_6_N_6_ monolayer, respectively (Figure 1). The observed bond distances are in good agreement with the already reported experimental and theoretical studies [70]. Each monolayer unit of C_6_N_6_ consists of covalently bonded s-triazine rings. The s-triazine rings contain nitrogen atoms, which makes the cavity of C_6_N_6_ highly electron rich. The diameter of the cavity in C_6_N_6_ is 5.46 Å (between two nitrogen atoms) [71]. The highly electron-rich nitrogenated cavity of C_6_N_6_ makes it a potential candidate for drug loading and delivery at target sites. The geometries of the selected anti-cancerous drugs (fluorouracil and nitrosourea; see Figure 1) were also optimized at the same level of theory (ωb97XD/6–31++G (d,p)). FU is an antimetabolite drug, which works by inhibiting essential biosynthetic processes or by being incorporated into macromolecules such as DNA and RNA. FU performs both functions. In the initial step, 5-FU exerts its anticancer effects through the inhibition of thymidylate synthase (TS) and the incorporation of its metabolites into DNA (see Figure 1) [72]. On other hand, nitrosourea is an alkylating agent with a long history in cancer treatment. Its mechanism of action is represented by the alkylation of DNA strands, which results in DNA damage and cellular death [73]. So, in both drugs, DNA is ultimately the common receptor.

**Figure 2:**
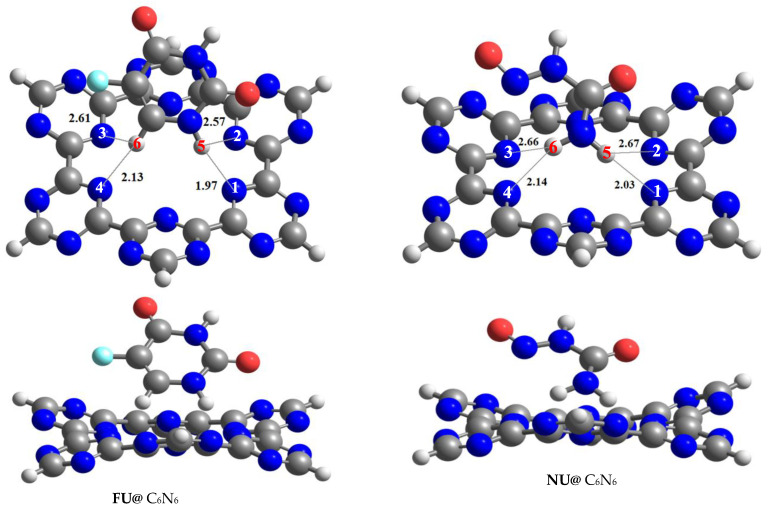
Top and tiled views of optimized geometries of most stable complexes of FU@C_6_N_6_ and NU@C_6_N_6_ at ωb97XD/6–31++G (d,p) level of theory.

Fluorouracil and nitrosourea were relaxed over C_6_N_6_ in different orientations to obtain the most stable Drug@C_6_N_6_ complexes for effective drug delivery at a specified target. The resultant most stable geometries of the Drug@C_6_N_6_ complexes are reported in Figure 2, while the less stable complexes of Drug@C_6_N_6_ are shown in the Supplementary Materials (Figure S1). Furthermore, the energies of the most stable Drug@C_6_N_6_ complexes are provided in Table 1, whereas all others are given in the Supplementary Materials (Table S1).

The most stable complexes of FU@C_6_N_6_ and NU@C_6_N_6_ resulted in adsorption energies of −28.14 kcal/mol and −26.57 kcal/mol, respectively. The stable geometries of both drug complexes were obtained as a result of the interaction of the H-atoms of the drugs with the N-atoms of the C_6_N_6_. This happened due to the electron-rich cavity of the C_6_N_6_ surface and the electrophilic nature of the H-atoms of the drug molecules. The adsorption energy values of the FU@C_6_N_6_ and NU@C_6_N_6_ complexes reveal that both drugs had strong binding with the C_6_N_6_ surface.

The adsorption energy of the most stable complex of the FU@C_6_N_6_ complex (−28.14 kcal/mol) was comparatively higher than that of the NU@C_6_N_6_ complex (−26.57 kcal/mol). The stable complex of the FU@C_6_N_6_ was obtained due to the shorter interaction distances of 1.97 Å (H5–N1) and 2.13 Å (H6–N3) between the H-atoms of the fluorouracil and the N-atoms of the C_6_N_6_. The rest of the H–N interaction distances noticed in the FU@C_6_N_6_ were 2.57 Å (H5–N2) and 2.61 Å (H6–N4). The interaction distances between the H-atoms of the NU and the N-atoms of the C_6_N_6_ were 2.03 Å (H5–N1) and 2.14 Å (H6–N4), which are slightly higher than those of the FU@C_6_N_6_ complex. The adsorption energies and interaction distances show the stability of the FU@C_6_N_6_ and NU@C_6_N_6_ complexes through physisorption, which is best for the C_6_N_6_ surface to act as a drug delivery platform.

### 3.2. Non-Covalent Interaction (NCI) Analysis

**Figure 3:**
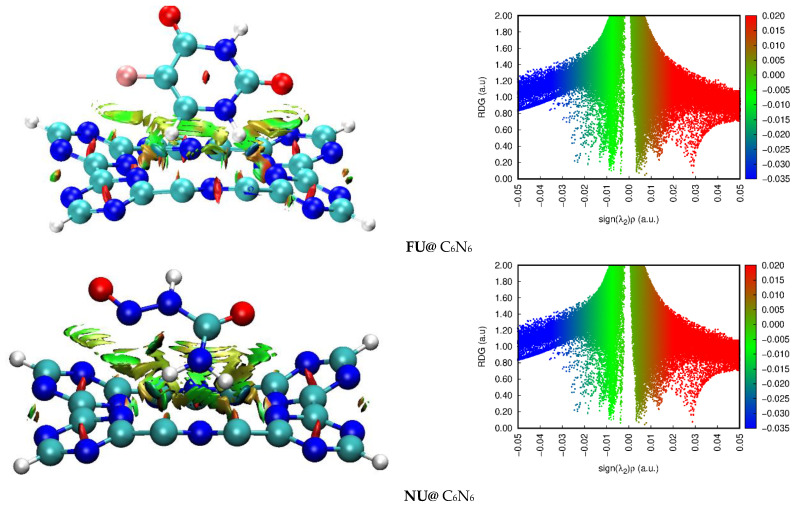
Three-dimensional isosurfaces and 2D RDG plots of FU@C_6_N_6_ and NU@C_6_N_6_ complexes at iso value of 0.002 a.u.

To differentiate between the electrostatic, van der Waals, and repulsive interactions between the drugs and C_6_N_6_, NCI analysis was performed. The results of the NCI analysis were displayed in 3D topological forms and 2D RDG plots. The topologies and RDG plots of the FU@C_6_N_6_ and NU@C_6_N_6_ complexes are presented in Figure 3. In the 3D topologies, the green and light brown patches show the stability of the FU@C_6_N_6_ and NU@C_6_N_6_ complexes by van der Waals interactions (see Figure 3). The red cylinder projections in the triazine rings of C_6_N_6_ indicate the ring’s steric strain. The thickness and area of green patches between the drugs and C_6_N_6_ are not the same, which reveals that the drug molecules (FU and NU) interacted with C_6_N_6_ via different energies.

In the case of the FU@C_6_N_6_, the green patches in the 3D plot are not specified between any two atoms of the drug and C_6_N_6_, but rather distributed among many atoms. The 3D topology of the FU@C_6_N_6_ reveals that the thick and wide green patches between the H-atoms of the FU and the N-atoms of the C_6_N_6_ caused stronger interactions and higher stability of the FU@C_6_N_6_ compared to the NU@C_6_N_6_ complex. Similarly, the mixture of the bluish-green spikes in the 2D RDG plot of the FU@C_6_N_6_ increases up to −0.02 a.u., whereas, in the case of the NU@C_6_N_6_, the green spikes are in the range of −0.02 to −0.01 a.u., which further justifies the higher stability of the FU@C_6_N_6_ complex than that of the NU@C_6_N_6_ complex. Therefore, the NCI analysis shows that both complexes were stabilized by van der Waals interactions. These interactions may facilitate the off-loading of the drug to the target site.

### 3.3. Quantum Theory of Atoms in Molecule (QTAIM) Analysis

Through QTAIM analysis, we can capture all the non-covalent interactions that are elusive to other methods. The strength of NCI mainly depends upon the sign and value of the electron density (ρ) and the Laplacian of the electron density (∇^2^ρ). In addition, the potential energy density (V), kinetic energy density, and their sum (which is described by the total energy density (H)) give valuable information about the BCPs. V/G is the ratio of the potential energy density (V) to the kinetic energy density (G). A V/G less than 1 indicates NCIs. In addition, an individual bond interaction energy of >−3.5 kcal/mol (negatively strong) shows shared shell interactions [74,75,76,77].

**Figure 4:**
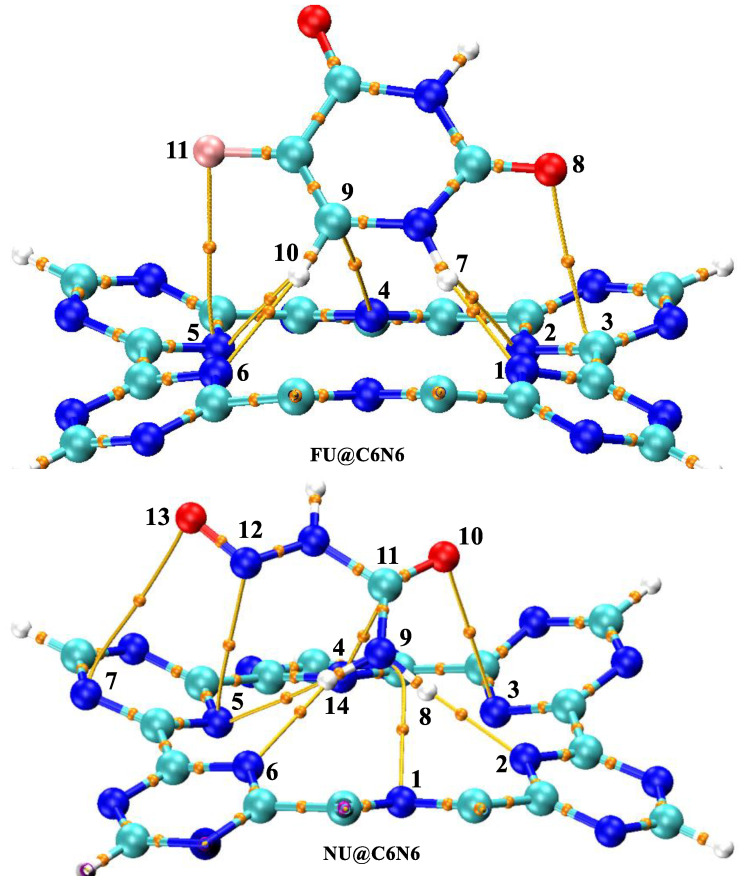
QTAIM analysis of FU@C_6_N_6_ and NU@C_6_N_6_.

The topologies obtained through the QTAIM analysis are presented in Figure 4, while the values of the BCP parameters are given in Table 2. In the QTAIM analysis of FU and NU, the numbers of BCPs observed were seven (07) and eight (08), respectively. These numbers actually represent the possible number of non-covalent interactions between the FU (and NU) with the C_6_N_6_. The values of the BCP parameters presented in Table 2 show that the stability of the FU@C_6_N_6_ complex happened not only through van der Waal interactions but also through the observed participation of electrostatic interactions. In the NU@C_6_N_6_ complex, the number of BCPs observed was eight, but the ρ and (∇^2^ρ) values were comparatively lower than those of the FU@C_6_N_6_ complex. Similarly, the rest of the BCP parameter values of the NU@C_6_N_6_ were also low compared to those of the FU@C_6_N_6_. Thus, the high stability of the FU complex was a result of the high values of ρ and (∇^2^ρ). These BCP parameters reveal that FU could easily be adsorbed on the carrier surface compared to NU. The results obtained through the QTAIM analysis are in good agreement with those of the interaction energies and NCI analysis.

### 3.4. Natural Bond Orbital (NBO) and Electron Density Differences (EDD) Analyses

**Figure 5:**
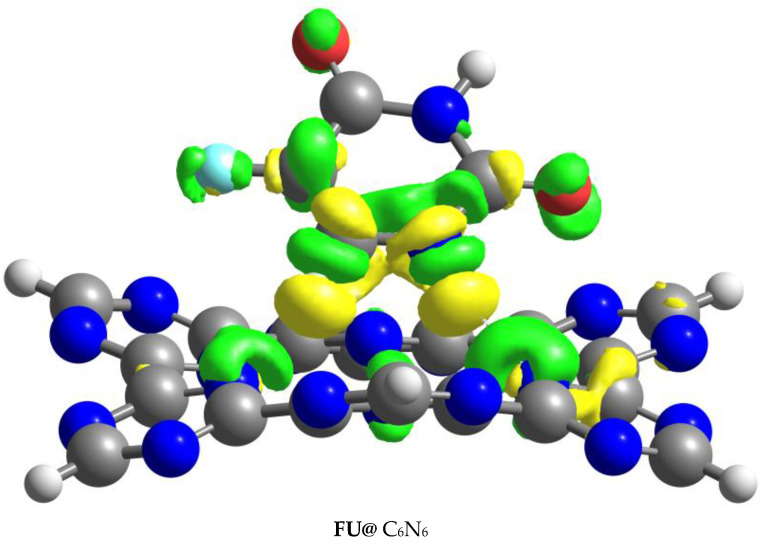

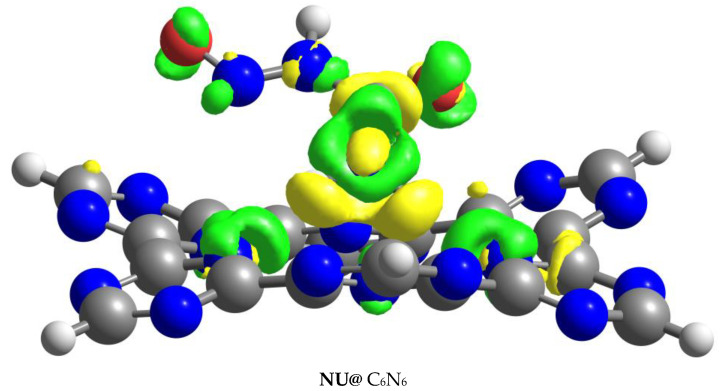
Isosurface plots of FU@C_6_N_6_ and NU@C_6_N_6_ complexes obtained by EDD analysis. Isovalue = 0.0014 a. u.

NBO and EDD analyses give valuable information about charge transfer in interacting systems. The magnitude of the charge transfer between the drug and C_6_N_6_ was explored through NBO analysis. The EDD was calculated by subtracting the sum of the drug and C_6_N_6_ charges from the charges of the Drug@C_6_N_6_ complex. The isosurfaces resulting from the EDD analysis consisted of two colors: green and yellow. The green isosurfaces indicate the accumulation of electron density, whereas the yellow surfaces show the depletion of electron density. So, the existence of both types of isosurfaces reveals the exchange of charges between the drug and C_6_N_6_. The isosurfaces executed through the EDD analysis are given in Figure 5. However, the values of charges obtained by the NBO analysis are shown in Table 3.

The NBO charge values of the FU and NU in the FU@C_6_N_6_ and NU@C_6_N_6_ complexes were −0.16 e^−^ and −0.02 e^−^, respectively. The signs of the NBO charges reflect that the charge was transferred from the C_6_N_6_ to the drug molecules. This happened due to the electron-rich cavity of the covalent triazine framework C_6_N_6_. A higher charge transfer value was noticed in the case of the FU@C_6_N_6_, which indicates stronger interactions between the FU and C_6_N_6_ compared to the complex of NU@C_6_N_6_. The higher NBO value on the FU@C_6_N_6_ complex was due to the presence of highly electronegative F and O atoms in the FU. In addition to this, the FU interacted with the C_6_N_6_ through shorter interaction distances compared to those of the NU atoms. The NBO values were verified by EDD analysis.

In the case of the FU@C_6_N_6_ and NU@C_6_N_6_ complexes, green isosurfaces mainly appeared on the N-atoms of the C_6_N_6_, showing an accumulation of electron density, whereas yellow isosurfaces appeared on the H-atoms, showing depletion of the electron density. The yellow isosurfaces on the H-atoms were due to their bonding with the O-atoms of the C_6_N_6_ and interactions with the N-atoms of the C_6_N_6_. This shows that charges were transferred from the triazine ring of the C_6_N_6_ to the FU and NU atoms. However, the drug atoms consist of both types of isosurfaces, which verifies the presence of electrophilic and nucleophilic ends on the drug. The NBO and EDD analyses indicate that the highest charge transfer was in the case of FU (−0.16 e^−^), which is in good agreement with the NCI, QTAIM, and interaction energy analyses. The higher charge exchange between the FU and C_6_N_6_ indicates the balanced loading capacity of the C_6_N_6_ to FU compared to that of NU.

### 3.5. Frontier Molecular Orbital (FMO) Analysis

FMO analysis was carried out to analyze the changes in the electronic properties of the C_6_N_6_ before and after complexations with the drug molecules. The energies of the highest occupied molecular orbital (HOMO), the lowest unoccupied molecular orbital (LUMO), and the HOMO-LUMO gaps (E_H-L_) of pristine C_6_N_6_ and Drug@C_6_N_6_ are given in Table 3.

**Figure 6:**
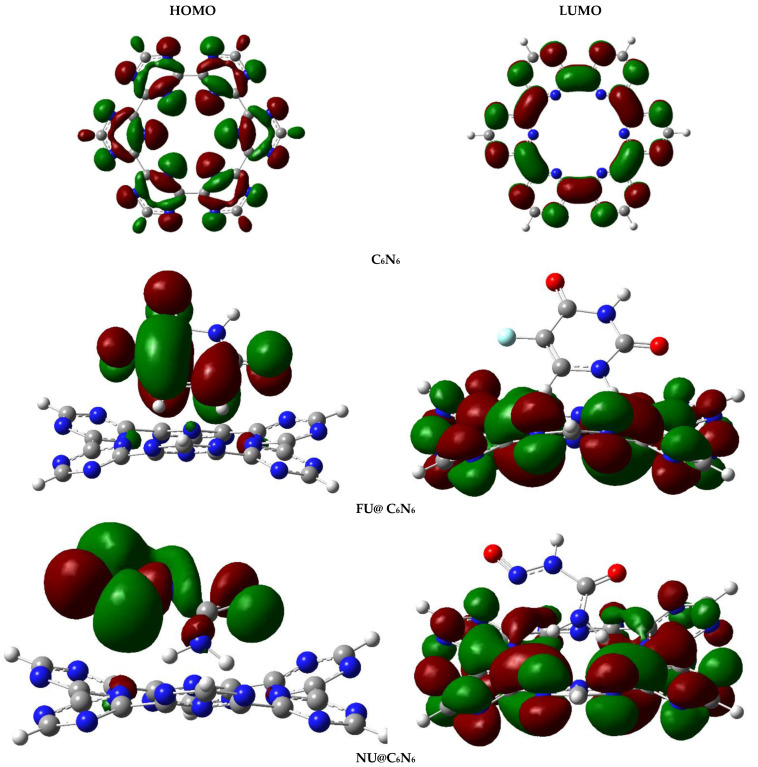
Orbital densities of FU@C_6_N_6_ and NU@C_6_N_6_ complexes obtained through FMO analysis (isovalue = 0.02 a.u.). Red isosurfaces show negative end of wavefunctions and green isosurfaces show positive wavefunctions.

The HOMO and LUMO energies of the C_6_N_6_ were −9.63 eV and −1.79 eV, respectively. The E_H-L_ gap of the C_6_N_6_ was 7.84 eV. A change in the E_H-L_ gap was noticed after the adsorption of drugs on the C_6_N_6_. Figure 6 depicts the orbital densities of the C_6_N_6_ and Drug@C_6_N_6_ complexes. The E_H-L_ gaps after the adsorption of FU and NU on the C_6_N_6_ were 6.71 eV (FU@C_6_N_6_) and 7.54 eV (NU@C_6_N_6_), respectively.

Decreases in the E_H-L_ gap were observed for the complexes of FU@C_6_N_6_ and NU@C_6_N_6_ compared to that of bare C_6_N_6_. The conductivity of the complexes depended on the E_H-L_ gap. Complexes with a low E_H-L_ gap showed better conductivity compared to those with a high E_H-L_ gap. This reveals that the complex of FU@C_6_N_6_ had better conductance than that of the NU@C_6_N_6_ complex. The substantial decrease in the E_H-L_ gap of the FU@CC_6_N_6_ happened due to a significant increase in the energy of the HOMO (from −9.63 to −8.63) and a decrease in the energy of LUMO (from −1.79 eV to −1.92 eV), respectively. The E_H-L_ gap for the NU@C_6_N_6_ complex was 7.54 eV, which was slightly lower than that of the bare C_6_N_6_ (7.84 eV). This happened due to a slight increase in the HOMO energy (−9.32 eV) while the LUMO energy remained almost unchanged (−1.78 eV).

The orbital isosurfaces for the complexes were also different compared to the pristine C_6_N_6_. The orbital densities of the FU@C_6_N_6_ and NU@C_6_N_6_ are presented in Figure 6. In the case of pristine C_6_N_6_, the HOMO densities were mainly distributed over the nitrogen atoms of the triazine rings, whereas the LUMO densities were on the carbon atoms. The orbital densities of the complexes show the charge transfer during the excitation from the HOMO to the LUMO. In the case of the FU@C_6_N_6_ complex, the HOMO density was majorly distributed over the FU, while a part of the HOMO density can also be seen in the C_6_N_6_. However, the LUMO was completely located on the C_6_N_6_, and the orbital density distribution pattern of the NU@C_6_N_6_ complex was slightly different from the FU@C_6_N_6_ complex. The maximum portion of the HOMO density was concentrated on the NU, while a small contribution was shown by the N-atoms of the C_6_N_6_, and the LUMO was fully distributed over the C_6_N_6_ surface. This shift of orbital densities from the FU (HOMO) to the C_6_N_6_ (LUMO) caused a significant decrease in the E_H-L_ gap. Thus, it can be concluded that the adsorption of FU caused an increase in the electrical conductivity of the C_6_N_6_ surface. This significant increase in the conductivity of the FU@C_6_N_6_ complex makes C_6_N_6_ a promising carrier for drug delivery through electrical therapy. The drug delivery mechanism of dexamethasone (DEX) from polydopamine/polypyrrole composites has also been reported on a conductivity basis [78,79] The results of the FMO analysis are in good agreement with those of the interaction energy, NCI, QTAIM, NBO, and EDD analyses.

## 4. Dipole Moment (µ) Analysis

The change in the dipole moment also helps to explain the solubility of the drug and its release at the target site. The µ of C_6_N_6_ COF is zero prior to the loading of drugs. This may happen due to the symmetry in the structure and the cancelation of the individual dipoles. The adsorption of FU and NU over C_6_N_6_ changed the µ values to 5.77 D and 3.25 D, respectively in the resultant complexes. The symmetry of the C_6_N_6_ was disturbed by the adsorption of the FU and NU on C_6_N_6_. This adsorption created new dipoles on the electron-withdrawing and -donating parts of complexes, where the drug and carrier surfaces interact through specific distances. The increase in the **µ** of complexes is essential for their solubility in an aqueous medium, which also helps the mobility of drugs in a living system. The substantial increase in the µ of the FU@C_6_N_6_ complex reveals the high affinity of FU for biological systems compared to NU. This shows that C_6_N_6_ can effectively release FU on a target site compared to NU. The results of the **µ** are consistent with the FMO, NBO, interaction energy, and NCI analyses.

### Comparison of Adsorption Energies of FU and NU with Different Surfaces

The adsorption energies of the FU@C_6_N_6_ (−28.14 kcal/mol) and NU@C_6_N_6_ (−27.54 kcal/mol) complexes were also compared with the already studied adsorption energies of FU and NU at different surfaces through different DFT tools. The reported adsorption energies of the drugs on different carriers are comparable to the values in the current study (FU@C_2_N; −26.3 kcal/mol and NU@C_2_N; −26.4 kcal/mol). In some cases, the adsorption energies were too high; FU@NaB40 fullerene and Ti-BNNT showed adsorption energies of −30.0 kcal/mol and −39.8 kcal/mole, respectively (see Table 4). These values of adsorption energies show that FU and NU can easily be off-loaded to the target site.

## 5. Drug Release

The drug release from the carrier surface to the target cell is one of the most important steps. The surrounding environment of a malignant cell environment usually has a pH of less than 6 compared to normal blood cells (7.35–7.45) [46]. Thus, we explored the pH effect on the complexes of FU@C_6_N_6_ and FU@C_6_N_6_. We carried out DFT simulations of drugs (FU@C_6_N_6_ and NU@C_6_N_6_) loaded on C_6_N_6_ in an acidic environment. In an acidic medium, we protonated the interacted ends (N-atoms) of the C_6_N_6_ with H^+^ and again relaxed the structure at the same level of theory. When comparing the FU@C_6_N_6_ and FU@C_6_N_6_ complexes in an acidic media, drastic decreases in the adsorption energies (from −28.14 to −1.15kcal/mole) and increases in the interaction distances (1.97 Å to 4.99 Å) were observed in the case of the FU@C_6_N_6_ complex. This indicates the easy off-loading of FU from the carrier (C_6_N_6_) to the target site compared to NU. In an acidic medium, a number of protons can also be attached to the other ends of the drug and surface. However, these protons may not affect the drug release at the target site.

## 6. Conclusions

To explore new drug delivery carriers, we used the covalent triazine framework C_6_N_6_ for FU and NU drugs through DFT simulations. Adsorption energies of −28.14 kcal/mol and −27.54 kcal/mol were observed in the most stable complexes of FU@C_6_N_6_ and NU@C_6_N_6_, respectively. The nature and strength of the non-covalent interactions were explored through NCI and QTAIM analyses. The outcomes of these analyses reveal that the stability of the FU@C_6_N_6_ and NU@C_6_N_6_ complexes was established through van der Waals interactions. The electronic properties of all the complexes were explored through NBO, EDD, and FMO analyses. Both the NBO and EDD analyses show an appreciable charge transfer between the drug and carrier. The FU@C_6_N_6_ complex had the highest charge transfer (−0.16 e^−^), while the NU@C_6_N_6_ complex had the lowest charge exchange (−0.02 e^−^). The E_H-L_ gaps of the 6.71 eV and 7.54 eV were observed for the FU@C_6_N_6_ and NU@C_6_N_6_ complexes, which were comparatively lower than that of the bare C_6_N_6_ (7.84 eV). The adsorption of the FU on the C_6_N_6_ caused a potential decrease in the E_H-L_ gap compared to that of the NU@C_6_N_6_. Thus, the loading of the FU on the C_6_N_6_ caused enhanced conductivity of the FU@C_6_N_6_ complex compared to that of the pristine C_6_N_6_. The results of the FMO analysis are consistent with those of the NBO, EDD, NCI, and QTAIM analyses. The drug release mechanisms were further studied through dipole moments and pH effects. Due to the low pH of malignant cells, simulations were performed in an acidic medium. The highest decrease in adsorption energy was observed for the FU@C_6_N_6_ complex in an acidic medium. These findings indicate that FU can easily be off-loaded from a carrier (C_6_N_6_) to a target site. Thus, it may be concluded that C_6_N_6_ is a better carrier of FU compared to NU for drug delivery.

## Figures and Tables

**Table 1:** Adsorption energies (kcal/mol) and geometric parameters of the most stable geometries of FU@C_6_N_6_ and NU@C_6_N_6_ complexes at ωb97XD/6-31++G (d,p) level of theory.

Drug@ C_6_N_6_	Drugs–C_6_N_6_	Adsorption Distance (Å)	Adsorption Energy (kcal/mol)
**FU@C_6_N_6_**	H5–N1	1.97	−28.14
	H5–N2	2.57	
	H6–N3	2.13	
	H6–N4	2.61	
**NU@C_6_N_6_**	H5–N1	2.03	−26.57
	H5–N2	2.67	
	H6–N3	2.66	
	H6–N4	2.14	

**Table 2:** Topological analysis of FU@C_6_N_6_ and NU@C_6_N_6_ obtained through QTAIM analysis.

Drugs@C_6_N_6_	Drug–C_6_N_6_	ρ	∇^2^ρ	G (r)	V (r)	H (r)	V(r)/G(r)	Eint (kcal/mole)
**FU@** **C_6_N_6_**	H7-N1	0.027	0.072	0.011	−0.010	0.0010	0.91	−3.17
	H7-N2	0.010	0.035	0.007	−0.006	0.0013	0.82	−1.91
	O8-C3	0.010	0.034	0.008	−0.007	0.0009	0.88	−2.09
	C4-N4	0.007	0.021	0.004	−0.004	0.0008	0.83	−1.15
	F11-N5	0.005	0.022	0.005	−0.004	0.0009	0.80	−1.15
	H10-N5	0.009	0.034	0.007	−0.005	0.0016	0.77	−1.63
	H10-N6	0.021	0.057	0.014	−0.014	0.0002	0.99	−4.34
**NU@** **C_6_N_6_**	H9-N1	0.008	0.032	0.007	−0.005	0.0014	0.79	−1.65
	H8-N2	0.024	0.067	0.017	−0.012	0.0041	0.75	−3.91
	O10-N3	0.009	0.030	0.007	−0.006	0.0006	0.90	−1.91
	C11-N4	0.008	0.030	0.006	−0.005	0.0013	0.80	−1.57
	N12-N5	0.009	0.027	0.006	−0.005	0.0007	0.88	−1.66
	O13-N7	0.004	0.015	0.003	−0.002	0.0006	0.79	−0.75
	H14-N5	0.007	0.026	0.006	−0.004	0.0011	0.80	−1.38
	H14-N6	0.008	0.032	0.007	−0.005	0.0014	0.79	−1.65

**Table 3:** Values of HOMO, LUMO, E_H-L_ gap, and NBO for FU@C_6_N_6_ and NU@C_6_N_6_ complexes.

Drug@C_6_N_6_	HOMO	LUMO	E_H-L_ gap (eV)	NBO (e^−^)
**C_6_N_6_**	−9.63	−1.79	7.84	
**FU@C_6_N_6_**	−8.63	−1.92	6.71	−0.16
**NU@C_6_N_6_**	−9.32	−1.78	7.54	−0.02

**Table 4:** Comparison of adsorption energies of FU@C_6_N_6_ and NU@C_6_N_6_ complexes with reported literature data on drug release.

DDS	DFT Functionals	Adsorption Energies (kcal/mol)
**FU@C_2_N**	M06-2x	−26.3 [80]
**NU@C_2_N**	M06-2x	−26.4 [80]
**FU@B_40_ fullerene**	PBE	−24.0 [81]
**FU@NaB_40_ fullerene**	PBE	−30.0 [81]
**FU@Ti-BNNT**	B3LY	−39.8 [82]
**NU@BC_3_**	PW91	−20.3 [83]
**NU@B_40_**	PBE0-D3	−25.1 [84]

## Data Availability

All data are provided in the manuscript and in the supplementary material.

## Supplementary Materials

The following supporting information can be downloaded at: https://www.mdpi.com/article/10.3390/ma15217425/s1, Figure S1. Top and side views of least stable complexes of FU@C6N6 (a–c) and NU@C6N6 (d–f); Table S1. Adsorption energies of least stable complexes of drug@C6N6.
