# Supplementary material for: Covalent Organic Framework (C6N6) as a Drug Delivery Platform for Fluorouracil to Treat Cancerous Cells: A DFT Study
Source: Materials (Basel). 2022 Oct 22;15(21):7425. doi: 10.3390/ma15217425 (PMC9654209; doi:10.3390/ma15217425)
Supplement: Supplementary file 1 [file materials-15-07425-s001.zip › materials-1968618-supplementary.pdf]

Supporting information

Covalent Organic Framework (C_6N_6) as a Drug Delivery Platform for Fluorouracil to Treat Cancerous Cells: A DFT Study

Mohammed A. Alkhalifah [1,*], Muhammad Yar [2], Imene Bayach [1], Nadeem S. Sheikh [3] and Khurshid Ayub [2,*]

[1] Department of Chemistry, College of Science, King Faisal University, Al-Ahsa 31982, Saudi Arabia
[2] Department of Chemistry, COMSATS University Islamabad, Abbottabad Campus, Abbottabad 22060, KPK, Pakistan
[3] Chemical Sciences, Faculty of Science, Universiti Brunei Darussalam, Jalan Tungku Link, Gadong BE1410, Brunei
* Correspondence: malkalifah@kfu.edu.sa (M.A.A.); khurshid@cuiatd.edu.pk (K.A.)

Table S1. Adsorption energies of least stable complexes of drug@C6N6.

**Drug@C6N6**	**Adsorption energies**
A	-28.13
B	-23.02
C	-28.10
D	-26.55
E	-19.35
F	-19.35

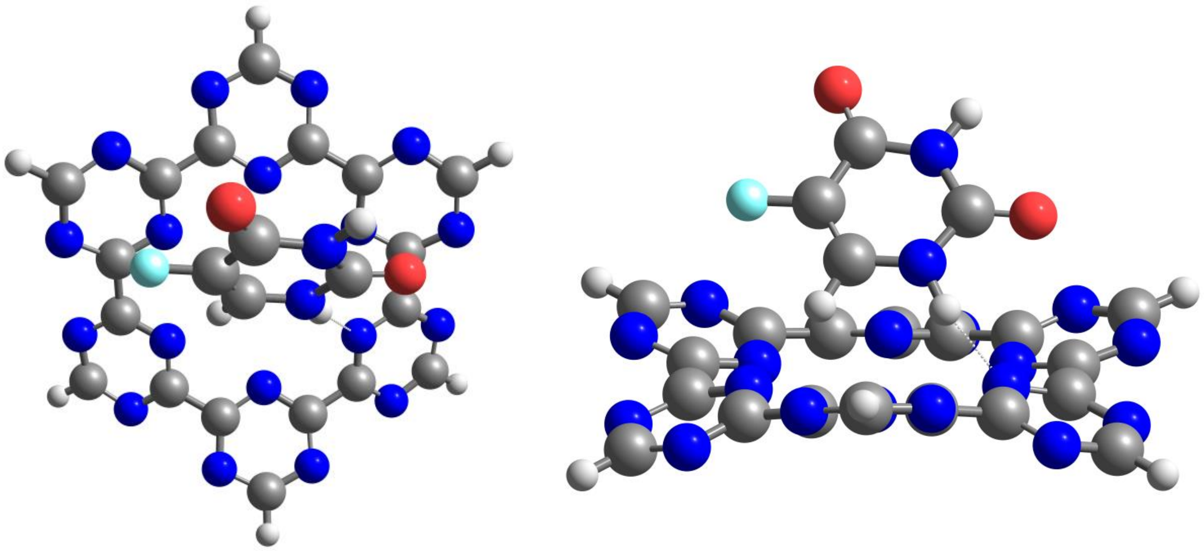

A

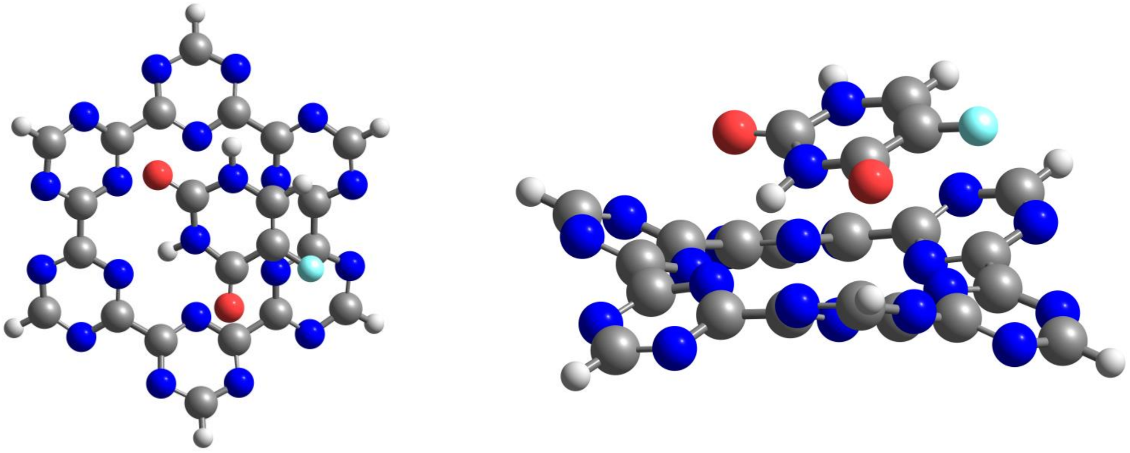

B

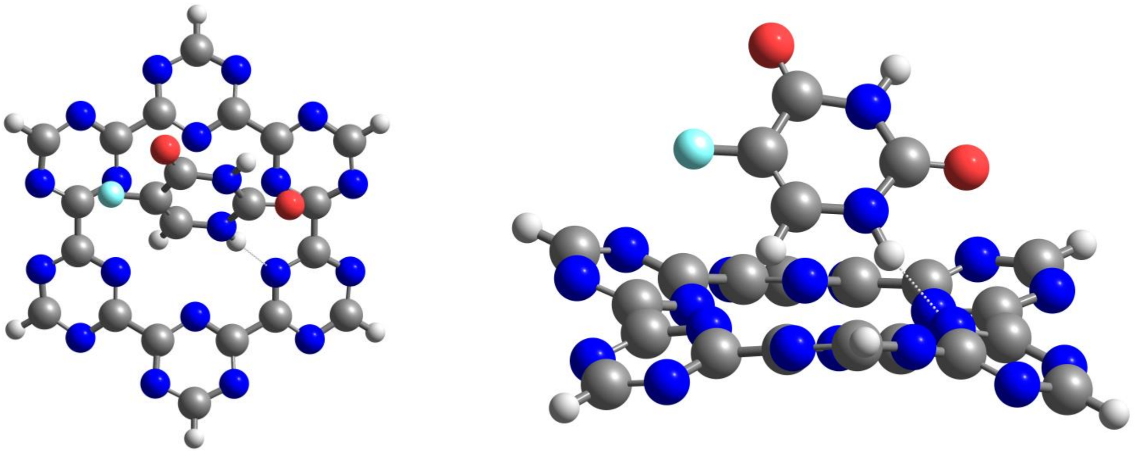

C

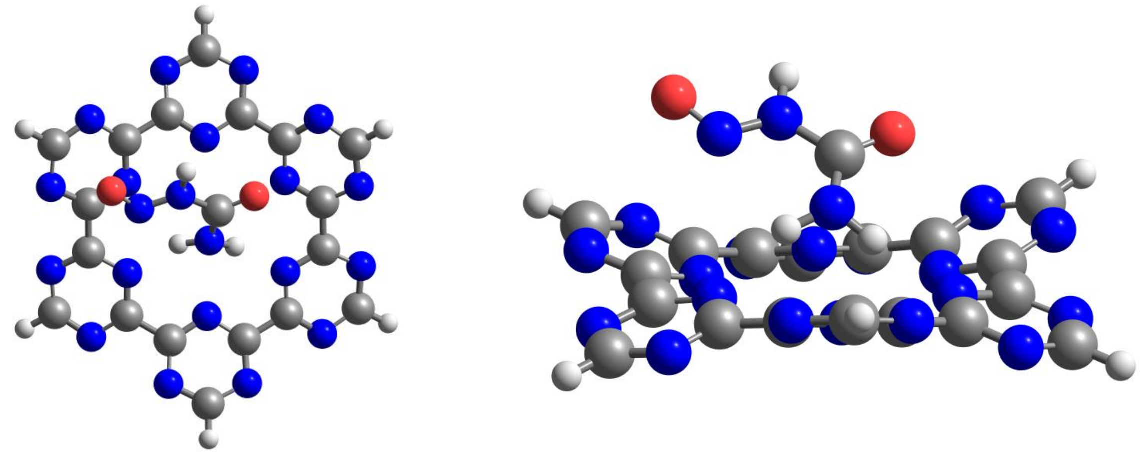

D

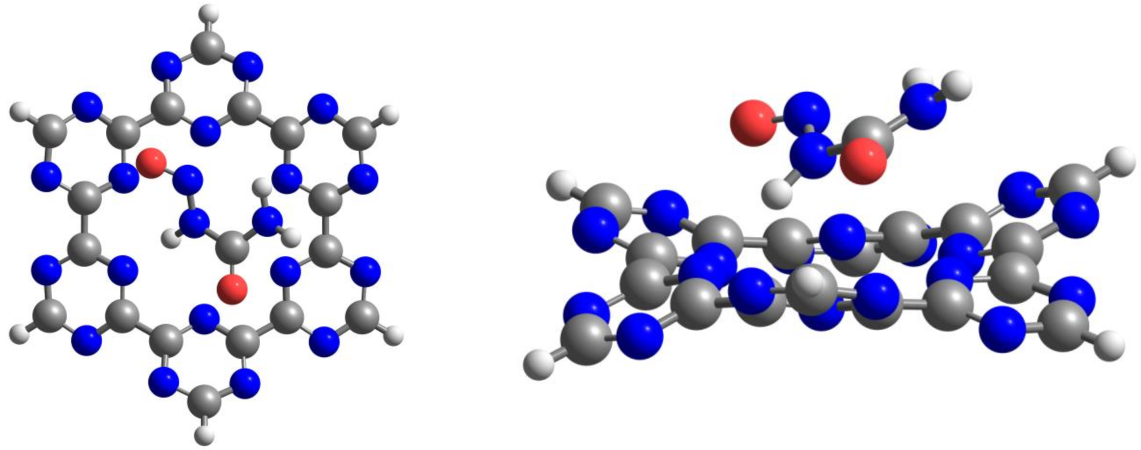

E

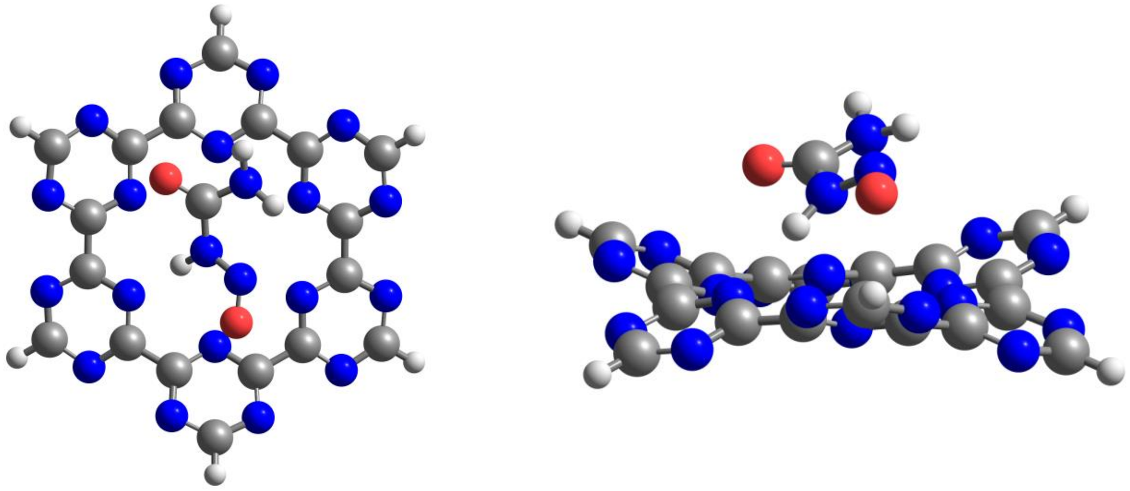

F

Figure S1. Top and side views of least stable complexes of FU@C6N6 (A–C) and NU@C6N6(D–F).
